# Reference genome of the leopard seal (*Hydrurga leptonyx*), a Southern Ocean apex predator

**DOI:** 10.3389/fgene.2025.1561273

**Published:** 2025-05-14

**Authors:** J. Canitz, S. S. Kienle, K. van der Linde, R. Borras-Chavez, E. S. Sperou, A. Leahy, S. Rivera, M. Autenrieth, J. I. Hoffman, C. A. Bonin

**Affiliations:** ^1^ Marine and Environmental Sciences Department, School of Science, Hampton University, Hampton, VA, United States; ^2^ Department of Natural Resources Science, College of Environmental Life Sciences, University of Rhode Island, Kingston, RI, United States; ^3^ Department of Biology, Baylor University, Waco, TX, United States; ^4^ School of Biological Sciences, University of Canterbury, Christchurch, New Zealand; ^5^ Jackson School of Geosciences, University of Texas at Austin, Austin, TX, United States; ^6^ Didactic of Biology, Institute of Biochemistry and Biology, Potsdam University, Potsdam, Germany; ^7^ Department of Evolutionary Population Genetics, University of Bielefeld, Bielefeld, Germany; ^8^ Center for Biotechnology (CeBiTec), Faculty of Biology, Bielefeld University, Bielefeld, Germany; ^9^ British Antarctic Survey, Cambridge, United Kingdom; ^10^ Joint Institute for Individualisation in a Changing Environment (JICE), Bielefeld University & University of Münster, Bielefeld, Germany; ^11^ Joint Institute for Individualisation in a Changing Environment (JICE), Bielefeld University & University of Münster, Münster, Germany

**Keywords:** leopard seal, reference genome, apex predator, PacBio sequencing, long read sequencing, Southern Ocean

## Introduction

Apex predators play a key role in maintaining ecosystem structure and functions (Ripple et al., 2014; Estes et al., 2016; Enquist et al., 2020). They exert top-down control on food webs, affecting nutrient and carbon cycles, modifying habitats, and regulating the spread of disease and invasive species (reviewed in Hammerschlag et al. (2019)). Hence, knowledge of the basic biology of apex predator species facilitates the prediction of their ecosystem impacts. However, it can be difficult to gather this knowledge for species that occupy remote habitats and have solitary lifestyles (Olivier et al., 2022).

The leopard seal (*Hydrurga leptonyx*) is an apex predator primarily found at low density (Southwell et al., 2008) in the Southern Ocean and subantarctic regions (Rogers, 2009; van der Linde et al., 2022; Borras-Chavez et al., 2024). Its diverse diet includes krill, cephalopods, fish and warm-blooded prey (e.g., seabirds and other seals; Siniff and Stone, 1985; Rogers, 2009), impacting different prey populations. The resulting predation pressure can be disproportionately high, which can significantly contribute to prey population collapse (Boveng et al., 1998; Schwarz et al., 2013; Krause et al., 2022). In the last decades leopard seals have been studied more intensively, especially in subantarctic areas, and more information about their foraging and movement patterns, physiology, morphometrics, and breeding behavior has been gathered (e.g., Rogers, 2017; Staniland et al., 2018; Krause et al., 2020; Kienle et al., 2022; Sperou et al., 2023; Kienle et al., 2024). Nevertheless, the relatively scarce information available for this species constrains our understanding of its role within the Antarctic ecosystem.

Genomic approaches can provide new insights into marine mammal ecology and evolution, such as understanding their spatiotemporal occurrence and abundance (Boyse et al., 2024), exploring their predator-prey dynamics (Visser et al., 2021), and investigating their demographic histories and population dynamics (Peart et al., 2020; Nebenführ et al., 2024). These methods are also growing in popularity due to the high-resolution they provide, leading to better-resolved results (Waldvogel et al., 2020). The affordability and accuracy of whole genome sequencing have advanced significantly with the development of technologies such as Illumina 10X, Oxford Nanopore, and PacBio HiFi, which are complemented by the ongoing improvement of bioinformatic tools. The use of whole genome sequencing also avoids common biases that are often encountered with specific genetic markers or genes. Studies aiming to investigate genetic variability within species or populations, or to characterize interspecific phylogenetic relationships, typically rely on reference genomes to facilitate the mapping of sequencing reads from multiple individuals (Fuentes-Pardo and Ruzzante, 2017; Theissinger et al., 2023). Reference genomes are also required for comparative genomics, transcriptomics, and epigenomics - scientific approaches that investigate species adaptation (Khudyakov et al., 2015; Yuan et al., 2021) and responses to changing environments (reviewed in Bernatchez et al. (2024)).

Nuclear reference genomes have been generated for only 17 pinniped species, representing approximately half of the taxonomic diversity within this group (Supplementary Table S1). Within the Monachinae subfamily, genome assemblies are currently available for four species: the Weddell seal (*Leptonychotes weddellii*), Northern elephant seal (*Mirounga angustirostris*), Southern elephant seal (*Miroun*g*a leonina*), and Hawaiian monk seal (*Neomonachus schauinslandi*; Supplementary Table S1). The Weddell seal is the only species from the Lobodontini tribe with a sequenced nuclear genome. This phylogenetic clade also includes the crabeater seal (*Lobodon carcinophaga*), Ross seal (*Ommatophoca rossii*), and leopard seal, all of which lack nuclear genome assemblies. Genomic resources for the leopard seal are currently limited to a mitochondrial genome (Arnason et al., 2006). Most genetic studies involving this species have employed classical genetic markers and targeted a broader context. Mitochondrial genes such as cytochrome b (*cyt b*), NADH dehydrogenase subunit 3 (*ND3*), and nuclear markers like the recombinase activating protein 1 (*RAG1*) have been sequenced in association with phylogenetic studies of Pinnipedia (Davis et al., 2004; Arnason et al., 2006; Fulton and Strobeck, 2010). Only three studies focusing on leopard seal genetics have been published to date: one uses microsatellite markers to infer population structure (Davis et al., 2008) and two are based on the hyper-variable region (D-Loop) of the mitochondrial control region to infer genetic diversity and historical demography (Hernández-Ardila et al., 2021; Bender et al., 2023).

In an era of constantly advancing sequencing technologies, the next logical step in leopard seal research is to apply genomic approaches to complement and enhance existing genetic data sets. Therefore, we present a high-quality reference genome for the leopard seal and evaluate its quality in comparison to the other four published genomes of the Monachinae subfamily.

## Materials and methods

### Sample collection

Organic tissue was collected during a necropsy of a stranded adult male leopard seal (Leopard Seal ID #: HLNZ-N013). The individual was found on 5 August 2023, at Kaitorete Beach, Christchurch, New Zealand (43° 50′0.781″S, 172° 34′43.0″E) and was not yet decomposed at the time of necropsy (6 August 2023). Tissue samples were stored in RNA later and frozen at −20°C. Sample collection was conducted under the permit number 63499-MAR (New Zealand), and samples were transported to the United States under permit NMFS permit #26767 (United States, valid from 07/11/2023 to 06/30/2028).

### Genome sequencing and assembly

Genomic DNA was extracted from kidney tissue using the Qiagen Genomic DNA Extraction Kit, following the manufacturer’s protocol. DNA quantity and quality were assessed using a Genomic DNA Screen Tape of a TapeStation System (Agilent) to ensure the presence of high-molecular-weight DNA, required for long-read HiFi sequencing (Supplementary Figure S1). Library preparation was performed using the SMRTbell Express Template Prep Kit 2.0, and sequencing was conducted on a PacBio Sequel IIe system with five SMRT cells. The raw reads were assembled using Hifiasm v0.15.4-r347 (Cheng et al., 2021). The resulting contigs were queried against the NCBI nucleotide (nt) database using BLAST to identify potential contaminants (Camacho et al., 2009). These results were analyzed with BlobTools v1.1.1 (Laetsch and Blaxter, 2017), and contaminants were identified and removed from the assembly. To further refine the assembly, haplotigs, and redundant contig overlaps were removed using purge_dups v1.2.5 (Guan et al., 2020). All laboratory procedures, including DNA extraction, HiFi sequencing, genome assembly, and contaminant analysis (including graphical visualization with BlobTools), were carried out by Cantata Bio/Dovetail Genomics (CA, United States).

### Genome quality assessment and synteny analysis

Cantata Bio/Dovetail Genomics conducted a quality assessment of the genome assembly. This included the calculation of N50, L50, N90, and L90 statistics, and a genome completeness analysis using BUSCO version 4.0.5 and the eukaryota_odb10 database (2020-09-10, number of species: 70, number of BUSCOs: 255; Manni et al., 2021). We calculated additional statistics such as GC content and the length of the longest contig (in bp) using the stats.sh script of bbtools v39.06 (Bushnell et al., 2017). To receive an additional level of genome completeness, we also repeated the BUSCO analysis with the carnivora_odb10 lineage dataset (Creation date: 2024-01-08, number of genomes: 12, number of BUSCOs: 14502; BUSCO version 5.7.0; Manni et al., 2021). Quality statistics of our *H. leptonyx* assembly were compared to published reference genomes of the Monachinae subfamily (*L. weddellii*: GCA_000349705.1, *M. angustirostris*: GCA_029215605.1, *M. leonina*: GCA_011800145.1, and *N. schauinslandi*: GCA_002201575.2). We conducted further quality and completeness assessments via synteny analyses with the high-quality reference genomes of closely related species. Our reference genome was aligned to the chromosome-level genome of a *N. schauinslandi* male using minimap2 v.2.28-r1209 (Li, 2018). Although minimap2 is generally used as a sequence mapping tool, the parameters were adjusted for pairwise whole genome alignment as suggested in the current software manual. We also aligned our reference genome to the scaffold-level genome of a female individual of the more closely related species *M. angustirostris* (Accession #: GCF029215605.1). Synteny plots were created using the JupiterPlot pipeline (https://github.com/JustinChu/JupiterPlot; 2024), which implements the plotting tool Circos (Krzywinski et al., 2009). For both plots, we chose to display a maximum number of 50 scaffolds/contigs (*maxScaff*) while showing only contigs that are larger than 0.05% of the total length of the leopard seal reference genome. The minimum scaffold/contig size of the reference species (*m*) was set to 5,000,000 bp, ensuring the inclusion of all chromosomes of *N. schauinslandi* and reducing the number of scaffolds for *M. angustirostris* to 17. Maximum gap length (*maxGap*), minimum mapping quality (*MAPQ*), and minimum bundle size (*minBundleSize*) were set to 20,000 bp, 55, and 100,000 bp, respectively. Finally, we assessed the amount of sequence repeats and identified repeat families in the reference genome using the software packages RepeatModeler v.2.0.5 and RepeatMasker v.4.1.2 with *de novo* default settings (Smit, Hubley and Green RepeatMasker Open-4.0. 2013-2015 http://www.repeatmasker.org).

## Results and discussion

### Quality assessment of the *Hydrurga leptonyx* reference genome

The leopard seal reference genome consists of 203 contigs with a total size of 2.4 Gbp. The genome assembly shows an average coverage of 86X and the number of contigs with a length sum corresponding to half of the genome (L50) being 9. The N50 value, which represents the sequence length of the shortest contig representing 50% of the genome, is 99.45 Mbp. The assembly has a GC content of 41.6%, and the BUSCO completeness scores are 94.9% and 98.2% using the eukaryote and carnivora datasets, respectively (Supplementary Files S1, S2). Based on these quality criteria, it is comparable to other pinniped reference genomes (Table 1). Moreover, it is an improvement over the only other currently available Lobodontini genome (*L. weddellii*; Noh et al., 2022) in terms of L50 and N50 values, and overall BUSCO completeness (Table 1). Thus, this leopard seal reference genome is likely the most complete Lobodontini genome to date.

**TABLE 1:** Quality statistics of the *Hydrurga leptonyx* reference genome compared to genome assemblies of Monachinae species (listed as NCBI reference sequence in November 2024).

	*H. leptonyx* (Leopard seal)	*L. weddellii* (Weddell seal)	*M. angustirostris* (Northern elephant seal)	*M. leonina* (Southern elephant seal)	*N. schauinslandi* (Hawaiian monk seal)
NCBI Refseq assembly ID	JBJQNM000000000^a^	GCF_000349705.1	GCF_029215605.1	GCF_011800145.1	GCF_002201575.2
Assembly level	Contig	Scaffold	Scaffold	Scaffold	Chromosome
Genome size [Gbp]	2.4	3.2	2.4	2.4	2.4
Number of scaffolds/contigs	203	16,710	497	1,114	8,094
Scaffold/Contig N50 [Mbp]	99.45	0.904	154.2	54.2	150.8
Scaffold/Contig L50	9	920	7	16	7
Longest scaffold/contig [Mbp]	210.9	12.7	215.9	111.6	214.7
Mean coverage	86X	82X	35.1X	100X	56X
GC content	41.6	41.5	42.0	41.5	41.5
BUSCO completeness (carnivora_odb10)	98.2%	84.4%	97.1%	97.6%	98.3%

^a^ NCBI accession number instead of NCBI Refseq assembly ID.

Approximately 35.91% of the reference genome is composed of repetitive elements. While some contigs consist almost exclusively of repetitive sequences, the proportion of repeats across the nine largest contigs ranges between 33.06% and 35.04% (L50 = 9; Supplementary Figure S2). These values are similar to the proportions reported for other phocid genomes (e.g., *M. leonina* = 41.51% Kim et al., 2020; *Phoca largha* = 35.83% Park et al., 2018). Out of the 35.91% repetitive sequences, 9.79% are retroelements (LINEs = 9.23%; LTRs = 0.55%), 0.43% are DNA transposons, 1.16% are simple repeats, 0.22% elements have low complexity and 24.32% are unclassified (Supplementary File S3). The high quality and completeness of this reference genome suggests that it will be eminently suitable for a variety of applications, both within and among species.

The genome assembly data and raw reads are deposited at the Genebank repository of the NCBI database (http://www.ncbi.nlm.nih.gov/) under the BioProject and BioSample ID PRJNA1194539 and SAMN45188358, respectively. The obtained genome assembly was submitted and registered under the NCBI GenBank accession number JBJQNM000000000. Raw long reads are publicly available at the NCBI Short Read Archive (SRA) under accession number SRR31619110.

### Synteny analyses

**FIGURE 1:**
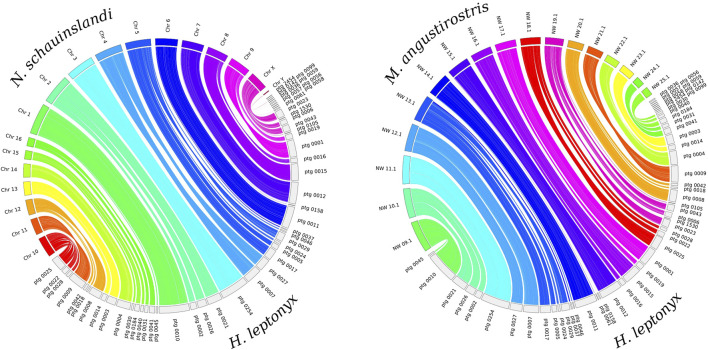
Synteny plots between the 50 largest contigs of the *Hydrurga leptonyx* reference genome and the 18 chromosomes of the *Neomonachus schauinslandi* reference genome (left) and the 17 largest scaffolds of the *Mirounga angustirostris* reference genome (right). The same contigs of *Hydrurga leptonyx* are colored equally in both plots.

The whole genome alignment reveals that the *H. leptonyx* genome assembly covers 94.85% and 93.86% of the *N. schauinslandi* and *M. angustirostris* reference genomes, respectively. In the synteny analysis, 45 contigs of the *H. leptonyx* reference genome are assigned to the 18 *N. schauinslandi* chromosomes, of which three contigs map to the Y chromosome (scaffold IDs: ptg0036, ptg0059, and ptg0142). With the same parameters, 42 of these 45 contigs also match the 17 longest scaffolds of the *M. angustirostris* reference genome (Figure 1). The difference in the total number of contigs revealing homology is due to the three contigs mapping to the Y chromosome of *N. schauinslandi*, as the *M. angustirostris* genome assembly belongs to a female. Furthermore, either individual long contigs or merged contigs correspond to each chromosome of *N. schauinslandi* (Figure 1). This is another indication of the high level of genome completeness, as chromosome numbers among pinnipeds are highly conserved (2n = 34 to 2n = 36, Arnason, 1974; Beklemisheva et al., 2020).

## Conclusion

Long-term observations of this apex predator are largely limited by the remoteness of the leopard seal’s habitat (i.e., pack-ice surrounding Antarctica) and their solitary nature. The resulting knowledge gaps about their basic biology and ecology prevent the effective incorporation of the species into ecosystem models. By presenting the first high-quality reference genome of the leopard seal, this study makes a significant step toward closing these gaps, providing a basic tool for future genomic analyses of the species’ molecular ecology and evolutionary history, as demonstrated for other pinnipeds (Yakupova et al., 2023; Hoffman et al., 2024; Hauser et al., 2024). In particular, genomic resources enable analyses of leopard seals’ fine-scale population structure and kinship and can be used for a more robust assessment of its past and present effective population size. Furthermore, this leopard seal reference genome serves as a valuable tool for examining genetic adaptations and the evolution of key traits. It also facilitates investigations on its adaptability and potential to respond to rapid environmental change, especially as leopard seals appear to be inherently vulnerable to ice loss in many areas of their global distribution (Bender et al., 2023; Borras-Chavez et al., 2024), and can be negatively affected by anthropogenic actions such as overharvesting at the lower levels of the food chain (Forcada et al., 2009).

## Data Availability

The datasets presented in this study can be found in online repositories. The names of the repository/repositories and accession number(s) can be found below: https://www.ncbi.nlm.nih.gov/genbank/, JBJQNM000000000; PRJNA1194539, SAMN45188358; https://www.ncbi.nlm.nih.gov/, SRR31619110.

## Supplementary material

SUPPLEMENTARY FIGURE S1 Results for the DNA quality by measuring with the Genomic Screen Tape of the Agilent TapeStation System. Average DNA fragment lengths are illustrated graphically and are listed in a table. This measurement ensures the presence of High Molecular Weight DNA.

SUPPLEMENTARY FIGURE S2 Sequence length and proportion of repeats for each contig of the *H. leptonyx* genome assembly. Contig lengths are indicated by the black line with the scale on the left and are sorted by size from left to right. The proportion of repeats per contig are shown in blue bars with the scale on the right. N50 and L50 are indicated by the horizontal and vertical red dashed lines, respectively.

SUPPLEMENTARY FILE S1 Cantata Bio/Dovetail Genomics HAM4300 Hifiasm Report for the H. leptonyx genome assembly. It consists of a summary table of genome statistics, graphical illustration of contaminants, a short description of material and methods as well as a list of references and software used for the preliminary analyses.

SUPPLEMENTARY FILE S2 Results of the BUSCO analysis using the H. leptonyx reference genome and the lineage data set carnivora_odb10 which includes 12 genomes and 14502 BUSCOs.

SUPPLEMENTARY FILE S3 Summary table of the repetitive elements found in the reference genome of H. leptonyx. The software packages RepeatModeler 2.0.5 and RepeatMasker version 4.1.2 were used to identify different repeat families to hard-mask the genome assembly.

SUPPLEMENTARY TABLE S1 List of all pinniped species with a published genome assembly (status 09/2024). Listed are Latin and common species names, the reference genome sequence identification number and its respective database, and a literature reference if available.

## References

[B1] Arnason U. (1974). Comparative chromosome studies in Pinnipedia. Hereditas 76 (2), 179–226. 10.1111/j.1601-5223.1974.tb01340.x 4135821

[B2] Arnason U. Gullberg A. Janke A. Kullberg M. Lehman N. Petrov E. A. (2006). Pinniped phylogeny and a new hypothesis for their origin and dispersal. Mol. Phylogenetics Evol. 41 (2), 345–354. 10.1016/j.ympev.2006.05.022 16815048

[B3] Beklemisheva V. R. Perelman P. L. Lemskaya N. A. Proskuryakova A. A. Serdyukova N. A. Burkanov V. N. (2020). Karyotype evolution in 10 pinniped species: variability of heterochromatin versus high conservatism of euchromatin as revealed by comparative molecular cytogenetics. Genes 11 (12), 1485. 10.3390/genes11121485 33321928 PMC7763226

[B4] Bender A. N. Krause D. J. Goebel M. E. Hoffman J. I. Lewallen E. A. Bonin C. A. (2023). Genetic diversity and demographic history of the leopard seal: a Southern Ocean top predator. PLoS One 18 (8), e0284640. 10.1371/journal.pone.0284640 37566609 PMC10420386

[B5] Bernatchez L. Ferchaud A. L. Berger C. S. Venney C. J. Xuereb A. (2024). Genomics for monitoring and understanding species responses to global climate change. Nat. Rev. Genet. 25 (3), 165–183. 10.1038/s41576-023-00657-y 37863940

[B6] Borras-Chavez R. Soteres R. L. Gómez-González G. Martínez F. Fernández-Ferrada N. Castillo-Aguilar M. (2024). Occurrence, residency, and habitat characterization of leopard seals in Chile. Front. Ecol. Evol. 12, 1448098. 10.3389/fevo.2024.1448098

[B7] Boveng P. L. Hiruki L. M. Schwartz M. K. Bengtson J. L. (1998). Population growth of Antarctic fur seals: limitation by a top predator, the leopard seal? Ecology 79 (8), 2863–2877. 10.1890/0012-9658(1998)079[2863:PGOAFS]2.0.CO;2

[B8] Boyse E. Robinson K. P. Beger M. Carr I. M. Taylor M. Valsecchi E. (2024). Environmental DNA reveals fine‐scale spatial and temporal variation of marine mammals and their prey species in a Scottish marine protected area. Environ. DNA 6 (4), e587. 10.1002/edn3.587

[B9] Bushnell B. Rood J. Singer E. (2017). BBMerge – accurate paired shotgun read merging via overlap. PLoS One 12 (10), e0185056. 10.1371/journal.pone.0185056 29073143 PMC5657622

[B10] Camacho C. Coulouris G. Avagyan V. Ma N. Papadopoulos J. Bealer K. (2009). BLAST+: architecture and applications. BMC Bioinforma. 10, 421–429. 10.1186/1471-2105-10-421 PMC2803857 20003500

[B11] Cheng H. Concepcion G. T. Feng X. Zhang H. Li H. (2021). Haplotype-resolved *de novo* assembly using phased assembly graphs with hifiasm. Nat. Methods 18 (2), 170–175. 10.1038/s41592-020-01056-5 33526886 PMC7961889

[B12] Davis C. S. Delisle I. Stirling I. Siniff D. B. Strobeck C. (2004). A phylogeny of the extant Phocidae inferred from complete mitochondrial DNA coding regions. Mol. Phylogenetics Evol. 33 (2), 363–377. 10.1016/j.ympev.2004.06.006 15336671

[B13] Davis C. S. Stirling I. A. N. Strobeck C. Coltman D. W. (2008). Population structure of ice‐breeding seals. Mol. Ecol. 17 (13), 3078–3094. 10.1111/j.1365-294X.2008.03819.x 18494764

[B14] Enquist B. J. Abraham A. J. Harfoot M. B. Malhi Y. Doughty C. E. (2020). The megabiota are disproportionately important for biosphere functioning. Nat. Commun. 11 (1), 699. 10.1038/s41467-020-14369-y 32019918 PMC7000713

[B15] Estes J. A. Heithaus M. McCauley D. J. Rasher D. B. Worm B. (2016). Megafaunal impacts on structure and function of ocean ecosystems. Annu. Rev. Environ. Resour. 41 (1), 83–116. 10.1146/annurev-environ-110615-085622

[B16] Forcada J. Malone D. Royle J. A. Staniland I. J. (2009). Modelling predation by transient leopard seals for an ecosystem-based management of Southern Ocean fisheries. Ecol. Model. 220 (12), 1513–1521. 10.1016/j.ecolmodel.2009.03.020

[B17] Fuentes‐Pardo A. P. Ruzzante D. E. (2017). Whole‐genome sequencing approaches for conservation biology: advantages, limitations and practical recommendations. Mol. Ecol. 26 (20), 5369–5406. 10.1111/mec.14264 28746784

[B18] Fulton T. L. Strobeck C. (2010). Multiple markers and multiple individuals refine true seal phylogeny and bring molecules and morphology back in line. Proc. R. Soc. B Biol. Sci. 277 (1684), 1065–1070. 10.1098/rspb.2009.1783 PMC2842760 19939841

[B19] Guan D. McCarthy S. A. Wood J. Howe K. Wang Y. Durbin R. (2020). Identifying and removing haplotypic duplication in primary genome assemblies. Bioinformatics 36 (9), 2896–2898. 10.1093/bioinformatics/btaa025 31971576 PMC7203741

[B20] Hammerschlag N. Schmitz O. J. Flecker A. S. Lafferty K. D. Sih A. Atwood T. B. (2019). Ecosystem function and services of aquatic predators in the Anthropocene. Trends Ecol. Evol. 34 (4), 369–383. 10.1016/j.tree.2019.01.005 30857757

[B21] Hauser S. Robinson S. Latch E. (2024). Genomic analysis of population history for Hawaiian monk seals. Endanger. Species Res. 53, 327–340. 10.3354/esr01308

[B22] Hernández-Ardila L. V. Barragán-Barrera D. C. Negrete J. Poljak S. Riet-Sapriza F. G. Caballero S. (2021). Insights into the genetic diversity of the leopard seal (*Hydrurga leptonyx*), inferred from mitochondrial DNA analysis, at Danco Coast, Antarctic Peninsula. Bol. Investig. Mar. Costeras-INVEMAR 50, 227–238. 10.25268/bimc.invemar.2021.50.suplesp.951

[B23] Hoffman J. I. Vendrami D. L. Hench K. Chen R. S. Stoffel M. A. Kardos M. (2024). Genomic and fitness consequences of a near-extinction event in the northern elephant seal. Nat. Ecol. and Evol. 8, 2309–2324. 10.1038/s41559-024-02533-2 39333394 PMC11618080

[B24] Khudyakov J. I. Preeyanon L. Champagne C. D. Ortiz R. M. Crocker D. E. (2015). Transcriptome analysis of northern elephant seal (*Mirounga angustirostris*) muscle tissue provides a novel molecular resource and physiological insights. BMC Genomics 16, 64–11. 10.1186/s12864-015-1253-6 25758323 PMC4328371

[B25] Kienle S. S. Bonin C. A. Gómez G. Goebel M. E. Donke M. Sperou E. S. (2024). First paired observations of sexual behavior and calls in wild leopard seals. Polar Biol. 47 (10), 1025–1037. 10.1007/s00300-024-03275-4

[B26] Kienle S. S. Goebel M. E. LaBrecque E. Borras-Chavez R. Trumble S. J. Kanatous S. B. (2022). Plasticity in the morphometrics and movements of an Antarctic apex predator, the leopard seal. Front. Mar. Sci. 9, 976019. 10.3389/fmars.2022.976019

[B27] Kim B. M. Lee Y. J. Kim J. H. Kim J. H. Kang S. Jo E. (2020). The genome assembly and annotation of the southern elephant seal *Mirounga leonina* . Genes 11 (2), 160. 10.3390/genes11020160 32028680 PMC7073746

[B28] Krause D. J. Bonin C. A. Goebel M. E. Reiss C. S. Watters G. M. (2022). The rapid population collapse of a key marine predator in the northern Antarctic Peninsula endangers genetic diversity and resilience to climate change. Front. Mar. Sci. 8, 796488. 10.3389/fmars.2021.796488

[B29] Krause D. J. Goebel M. E. Kurle C. M. (2020). Leopard seal diets in a rapidly warming polar region vary by year, season, sex, and body size. BMC Ecol. 20, 32–15. 10.1186/s12898-020-00300-y 32493329 PMC7271520

[B30] Krzywinski M. Schein J. Birol I. Connors J. Gascoyne R. Horsman D. (2009). Circos: an information aesthetic for comparative genomics. Genome Res. 19 (9), 1639–1645. 10.1101/gr.092759.109 19541911 PMC2752132

[B31] Laetsch D. R. Blaxter M. L. (2017). BlobTools: interrogation of genome assemblies. F1000Research 6, 1287. 10.12688/f1000research.12232.1

[B32] Li H. (2018). Minimap2: pairwise alignment for nucleotide sequences. Bioinformatics 34 (18), 3094–3100. 10.1093/bioinformatics/bty191 29750242 PMC6137996

[B33] Manni M. Berkeley M. R. Seppey M. Simão F. A. Zdobnov E. M. (2021). BUSCO update: novel and streamlined workflows along with broader and deeper phylogenetic coverage for scoring of eukaryotic, prokaryotic, and viral genomes. Mol. Biol. Evol. 38 (10), 4647–4654. 10.1093/molbev/msab199 34320186 PMC8476166

[B34] Nebenführ M. Arnason U. Janke A. (2024). Whole-genome re-sequencing of the Baikal seal and other phocid seals for a glimpse into their genetic diversity, demographic history, and phylogeny. GigaByte 2024, gigabyte142. 10.46471/gigabyte.142 39610871 PMC11602651

[B35] Noh H. J. Turner-Maier J. Schulberg S. A. Fitzgerald M. L. Johnson J. Allen K. N. (2022). The Antarctic Weddell seal genome reveals evidence of selection on cardiovascular phenotype and lipid handling. Commun. Biol. 5 (1), 140. 10.1038/s42003-022-03089-2 35177770 PMC8854659

[B36] Olivier C. A. Schradin C. Makuya L. (2022). Global change and conservation of solitary mammals. Front. Ecol. Evol. 10, 906446. 10.3389/fevo.2022.906446

[B37] Park J. Y. Kim K. Sohn H. Kim H. W. An Y. R. Kang J. H. (2018). Deciphering the evolutionary signatures of pinnipeds using novel genome sequences: the first genomes of *Phoca largha, Callorhinus ursinus*, and *Eumetopias jubatus* . Sci. Rep. 8 (1), 16877. 10.1038/s41598-018-34758-0 30442995 PMC6237890

[B38] Peart C. R. Tusso S. Pophaly S. D. Botero-Castro F. Wu C. C. Aurioles-Gamboa D. (2020). Determinants of genetic variation across eco-evolutionary scales in pinnipeds. Nat. Ecol. and Evol. 4 (8), 1095–1104. 10.1038/s41559-020-1215-5 32514167

[B39] Ripple W. J. Estes J. A. Beschta R. L. Wilmers C. C. Ritchie E. G. Hebblewhite M. (2014). Status and ecological effects of the world’s largest carnivores. Science 343 (6167), 1241484. 10.1126/science.1241484 24408439

[B40] Rogers T. L. (2009). “Leopard seal: hydrurga leptonyx,” in Encyclopedia of marine mammals. Editors Perrin W. F. Wuersig B. Thewissen J. G. M. 2nd Edn: Academic Press, 673–674. 10.1016/B978-0-12-373553-9.00155-3

[B41] Rogers T. L. (2017). Calling underwater is a costly signal: size-related differences in the call rates of Antarctic leopard seals. Curr. Zool. 63 (4), 433–443. 10.1093/cz/zox028 29492003 PMC5804190

[B42] Schwarz L. K. Goebel M. E. Costa D. P. Kilpatrick A. M. (2013). Top‐down and bottom‐up influences on demographic rates of Antarctic fur seals *Arctocephalus gazella* . J. Animal Ecol. 82 (4), 903–911. 10.1111/1365-2656.12059 23444975

[B43] Siniff D. B. Stone S. (1985). “The role of the leopard seal in the tropho-dynamics of the Antarctic marine ecosystem,” in In Antarctic nutrient cycles and food webs (Berlin, Heidelberg: Springer Berlin Heidelberg), 555–560. 10.1007/978-3-642-82275-9_75

[B44] Southwell C. Paxton C. G. Borchers D. Boveng P. Rogers T. de la Mare W. K. (2008). Uncommon or cryptic? Challenges in estimating leopard seal abundance by conventional but state-of-the-art methods. Deep Sea Res. 55 (4), 519–531. 10.1016/j.dsr.2008.01.005

[B45] Sperou E. S. Crocker D. E. Borras-Chavez R. Costa D. P. Goebel M. E. Kanatous S. B. (2023). Large and in charge: cortisol levels vary with sex, diet, and body mass in an Antarctic predator, the leopard seal. Front. Mar. Sci. 10, 1179236. 10.3389/fmars.2023.1179236

[B46] Staniland I. J. Ratcliffe N. Trathan P. N. Forcada J. (2018). Long term movements and activity patterns of an Antarctic marine apex predator: the leopard seal. PLoS One 13 (6), e0197767. 10.1371/journal.pone.0197767 29870541 PMC5988266

[B47] Theissinger K. Fernandes C. Formenti G. Bista I. Berg P. R. Bleidorn C. (2023). How genomics can help biodiversity conservation. Trends Genet. 39 (7), 545–559. 10.1016/j.tig.2023.01.005 36801111

[B48] van der Linde K. Visser I. N. Bout R. Krause D. J. Forcada J. Siniff D. (2022). A review of leopard seal (*Hydrurga leptonyx*) births and pups using a standardised age-class classification system. Polar Biol. 45 (7), 1193–1209. 10.1007/s00300-022-03053-0

[B49] Visser F. Merten V. J. Bayer T. Oudejans M. G. De Jonge D. S. W. Puebla O. (2021). Deep-sea predator niche segregation revealed by combined cetacean biologging and eDNA analysis of cephalopod prey. Sci. Adv. 7 (14), eabf5908. 10.1126/sciadv.abf5908 33789903 PMC8011969

[B50] Waldvogel A. M. Feldmeyer B. Rolshausen G. Exposito-Alonso M. Rellstab C. Kofler R. (2020). Evolutionary genomics can improve prediction of species’ responses to climate change. Evol. Lett. 4 (1), 4–18. 10.1002/evl3.154 32055407 PMC7006467

[B51] Yakupova A. Tomarovsky A. Totikov A. Beklemisheva V. Logacheva M. Perelman P. L. (2023). Chromosome-length assembly of the baikal seal (*Pusa sibirica*) genome reveals a historically large population prior to isolation in lake baikal. Genes 14 (3), 619. 10.3390/genes14030619 36980891 PMC10048373

[B52] Yuan Y. Zhang Y. Zhang P. Liu C. Wang J. Gao H. (2021). Comparative genomics provides insights into the aquatic adaptations of mammals. Proc. Natl. Acad. Sci. 118 (37), e2106080118. 10.1073/pnas.2106080118 34503999 PMC8449357

