# Supplementary material for: Reference genome of the leopard seal (Hydrurga leptonyx), a Southern Ocean apex predator
Source: Front Genet. 2025 May 14;16:1561273. doi: 10.3389/fgene.2025.1561273 (PMC12118156; doi:10.3389/fgene.2025.1561273)
Supplement: Supplementary file 1 [file Table1.pdf]

Supplementary Table S1: List of all pinniped species with a published genome assembly (status 09/2024). Listed are latin and common species names, the reference genome sequence identification number and its respective database, and a literature reference if available.

**Latin name**	**Common name**	**Higher taxonomic level**	**Reference ID/RefSeq ID (Database)**	**Assembly reference**
Zalophus californianus	California Sea Lion	Otariidae	GCF_009762305.2 (NCBI)	Vertebrate Genome Project/G10X (2020)
Otaria byronia	South American Sea Lion	Otariidae	CNP000758 (China National GeneBank Nucleotide Sequence Archive)	Yuan et al. 2021 (PNAS)
Eumetopias jubatus	Stellar Sea Lion	Otariidae	GCF_004028035.1 (NCBI)	Canada’s Genome Enterprice/BC Cancer Agency (2019)
Arctocephalus australis	South American Fur Seal	Otariidae	CNP000758 (China National GeneBank Nucleotide Sequence Archive)	Yuan et al. 2021 (PNAS)
Arctocephalus gazella	Antarctic Fur Seal	Otariidae	GCA_040869175.1 (NCBI)	University Bielefeld (2024), Hench et al. (2024)
Callorhinus ursinus	Northern fur seal	Otariidae	GCF_003265705.1 (NCBI)	Canada's Genomics Enterprise/BC Cancer Agency (2018)
Phoca vitulina	Habor Seal	Phocidae/ Phocinae	GCF_004348235.1 (NCBI)/ GSC_Hseal_1.0_HiC (DNA Zoo)	Canada’s Genome Enterprice/BC Cancer (2019)
Phoca largha	Spotted Seal	Phocidae/ Phocinae	Phoca_largha_HiC (DNA Zoo)	Baylor College of Medicine/DNA Zoo (2024)
Pusa sibirica	Baikal Seal	Phocidae/Phocina	GCA_028975605.1 (NCBI)	Institute for Molecular and Celllular Biology (2023)
Pusa hispida saimensis	Saimaa Ringed Seal	Phocidae/ Phocinae	GCA_947044825.1 (NCBI)	Institute of Biotechnology, University of Helsinki (2023)
Arctocephalus townsendi	Guadalupe Fur Seal	Otariidae	GCA_028646355.1 (NCBI)/ Arctocephalus_townsendi_HiC.assembly (DNA Zoo)	Baylor College of Medicine/DNA Zoo (2023)
Mirounga angustirostris	Northern Elephant Seal	Phocidae/ Monachinae	GCF_029215605.1 (NCBI)	University of California, Los Angeles (UCLA, 2023)
Mirounga leonina	Southern Elephant Seal	Phocidae/ Monachinae	GCF_011800145.1 (NCBI)	Earth Biogenome Project/Julie Ann Wrigley Global Futures Laboratory – Arizona State University (2020)
Halichoerus grypus	Gray Seal	Phocidae/ Phocinae	GCF_012393455.1 (NCBI)/ Halichoerus_grypus_HiC (DNA Zoo)	Cummings School of Veterinary Medicine/Tufts University (2020) and Baylor College of Medicine/DNA Zoo (2023)
Neomonachus schauinslandi	Hawaiian Monk Seal	Phocidae/ Monachinae	GCF_002201575.2 (NCBI)	John Hopkins University (2021)/Mohr et al. (2022)

Leptonychotes weddellii	Weddell Seal	Phocidae/ Monachinae	GCF_000349705.1 (NCBI)	Broad Institute (2013)
Odobenus rosmarus	Pacific Walrus	Obobenidae	GCF_000321225.1 (NCBI)	Marine Mammals (2013)/ Foote et al. (2015)

References:

Clawson, H., Lee, B.T., Raney, B.J., Barber, G.P., Casper, J., Diekhans, M., Fischer, C., Gonzalez, J.N., Hinrichs, A.S., Lee, C.M. and Nassar, L.R., 2023. GenArk: towards a million UCSC genome browsers. *Genome Biology*, *24*(1), p.217.

Foote, A.D., Liu, Y., Thomas, G.W., Vinař, T., Alföldi, J., Deng, J., Dugan, S., van Elk, C.E., Hunter, M.E., Joshi, V., Khan, Z. (2015). Convergent evolution of the genomes of marine mammals. *Nature genetics*, *47*(3), pp.272-275. https://doi.org/10.1038/ng.3198

Hench, K., Vendrami, D.L., Forcada, J., Hoffman, J.I. (2024). Refinement of the Antarctic fur seal (*Arctocephalus gazella*) reference genome increases continuity and completeness. *G3: Genes*, *Genomes*, *Genetics*, *14*(11), jkae179. https://doi.org/10.1093/g3journal/jkae179

Mohr, D.W., Gaughran, S.J., Paschall, J., Naguib, A., Pang, A.W.C., Dudchenko, O., Aiden, E.L., Church, D.M., Scott, A.F. (2022). A chromosome-length assembly of the Hawaiian monk seal (*Neomonachus schauinslandi*): A history of "Genetic Purging" and genomic stability. *Genes*, *13*(7), p.1270. https://doi.org/10.3390/genes13071270

Yuan, Y., Zhang, Y., Zhang, P., Liu, C., Wang, J., Gao, H., Hoelzel, A.R., Seim, I., Lv, M., Lin, M., Dong, L. (2021). Comparative genomics provides insights into the aquatic adaptations of mammals. *Proceedings of the National Academy of Sciences*, *118*(37), e2106080118. https://doi.org/10.1073/pnas.2106080118
