# Supplementary material for: Reference genome of the leopard seal (Hydrurga leptonyx), a Southern Ocean apex predator
Source: Front Genet. 2025 May 14;16:1561273. doi: 10.3389/fgene.2025.1561273 (PMC12118156; doi:10.3389/fgene.2025.1561273)
Supplement: Supplementary file 2 [file Supplementaryfile1.pdf]

Supplementary File S1: Cantata Bio/Dovetail Genomics HAM4300 Hifiasm Report for the *H. leptonyx* genome assembly. It consists of a summary table of genome statistics, graphical illustration of contaminants, a short description of material and methods as well as a list of references and software used for the preliminary analyses.

HAM4300 - Hifiasm Report

January 20, 2024

Hydrurga leptonyx

Carolina Lewallen

Hampton University

Contents

- Input Summary
- Overview
- BUSCO
- Blobplot Image
- Materials and Methods
- References
- Software Versions

Input Data

LIMS ID	Number of Reads	Bp (Gb)	Genome Size Estimate	Coverage
HAM4300	14,275,595	206.9	2.4	86x

Overview

Assembly	Total Length (bp)	N50	L50	N90	L90
Hifiasm Assembly	2,725,942,073	89,118,040	11	1,673,456	52
Primary Filtered Assembly	2,455,564,834	99,454,515	9	31,951,825	27

BUSCO

Assembly	Complete BUSCOs (C)	Complete and single-copy BUSCOs (S)	Complete and duplicated BUSCOs (D)	Fragmented BUSCOs (F)	Missing BUSCOs (M)	Total BUSCO groups searched
Hifiasm Assembly	247 (96.86%)	209 (81.96%)	38	2	6	255
Primary Filtered Assembly	242 (94.90%)	233 (91.37%)	9	7	6	255

- BUSCO version is: 4.0.5
- The lineage dataset is: eukaryota_odb10 (Creation date: 2020-09-10, number of species: 70, number of BUSCOs: 255)

Blobplot Image

blob_plot.blob.blobDB.json.bestsum.phylum.p8.span.100.blobplot.bam0

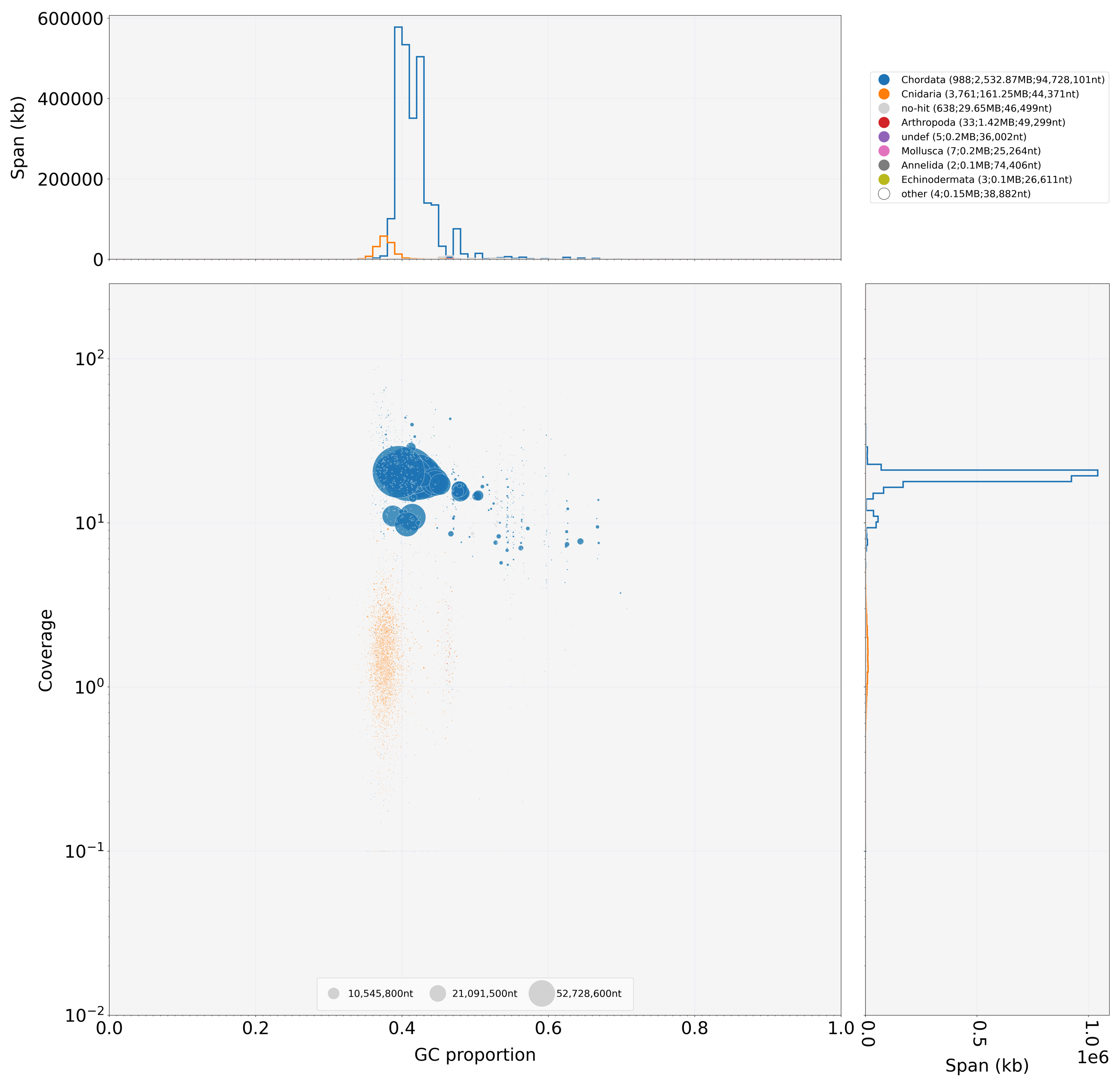

Materials and Methods

206.9 gigabase-pairs of PacBio CCS reads were used as an input to Hifiasm[1] v0.15.4-r347 with default parameters.

Blast results of the Hifiasm output assembly (`hifiasm.p_ctg.fa`) against the nt database were used as input for blobtools[2] v1.1.1 and scaffolds identified as possible contamination were removed from the assembly (`filtered.asm.cns.fa`). Finally, purge_dups[3] v1.2.5 was used to remove haplotigs and contig overlaps (`purged.fa`).

For additional software version information, please see our conda environment below.

References

1. Cheng, H., Concepcion, G.T., Feng, X., Zhang, H., Li H. Haplotype-resolved de novo assembly using phased assembly graphs with hifiasm. Nat Methods 18, 170-175 (2021). https://doi.org/10.1038/s41592-020-01056-5

2. Laetsch DR, and Blaxter ML. BlobTools: Interrogation of genome assemblies [version 1; peer review: 2 approved with reservations]. F1000Research 2017, 6:1287 https://doi.org/10.12688/f1000research.12232.1

3. Guan D, McCarthy SA, Wood J, Howe K, Wang Y, Durbin R. Identifying and removing haplotypic duplication in primary genome assemblies. Bioinformatics. 2020 May 1;36(9):2896-2898. doi: 10.1093/bioinformatics/btaa025. PMID: 31971576; PMCID: PMC7203741.

Software Versions

Package	Version
awscli	1.20.0
bioawk	1.0
blas	1.0
blast	2.13.0
blobtools	1.1.1
minimap2	2.21
numpy	1.19.1
pandas	1.1.3
pip	20.2.4
pyqt	5.9.2
pysam	0.15.4
python	3.7.6
qt	5.9.7
samtools	1.9
wtdbg	2.5
