# Supplementary figures and images for: Reference genome of the leopard seal (Hydrurga leptonyx), a Southern Ocean apex predator
Source: Front Genet. 2025 May 14;16:1561273. doi: 10.3389/fgene.2025.1561273 (PMC12118156; doi:10.3389/fgene.2025.1561273)

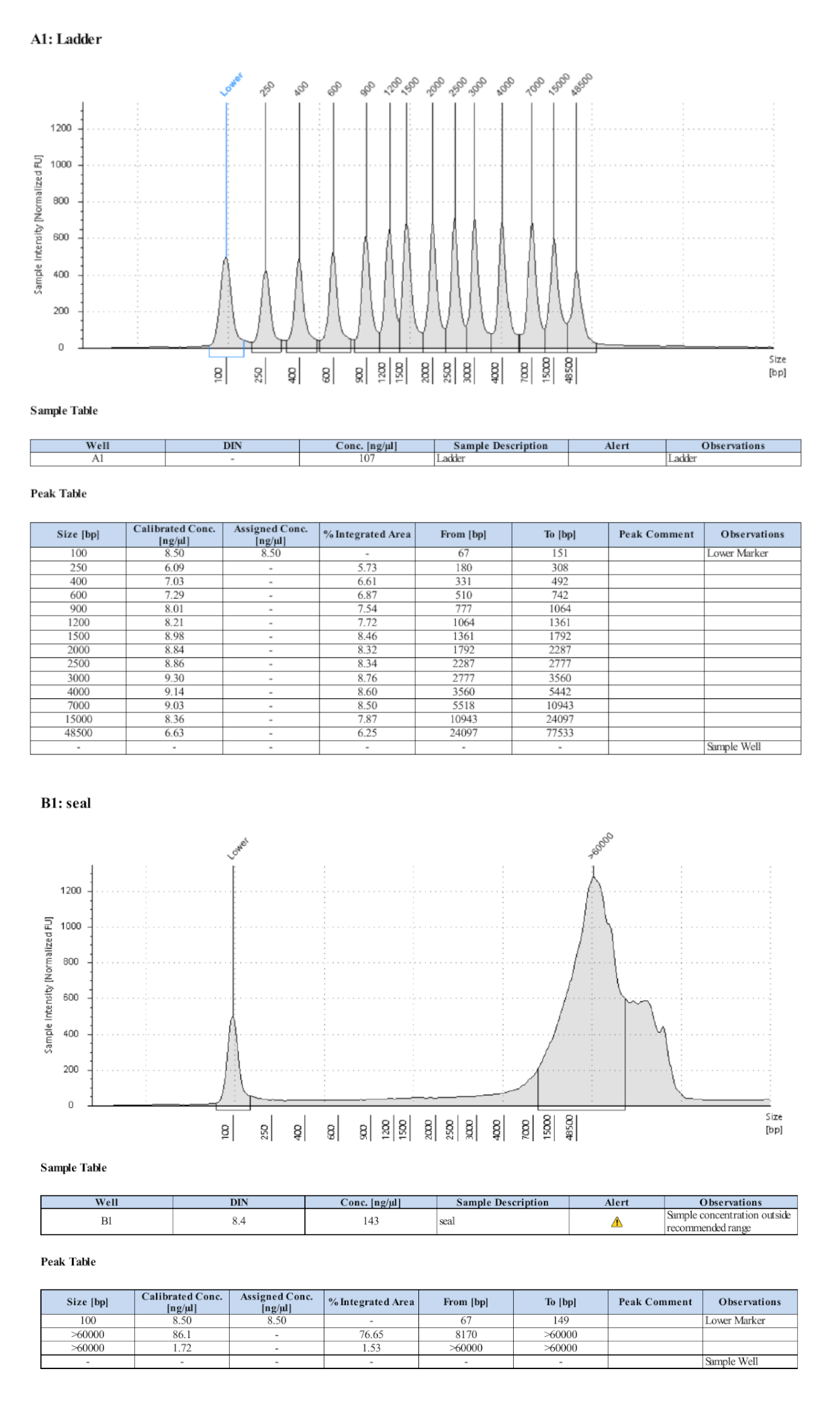

Supplement: Supplementary file 3 [file Image2.png]

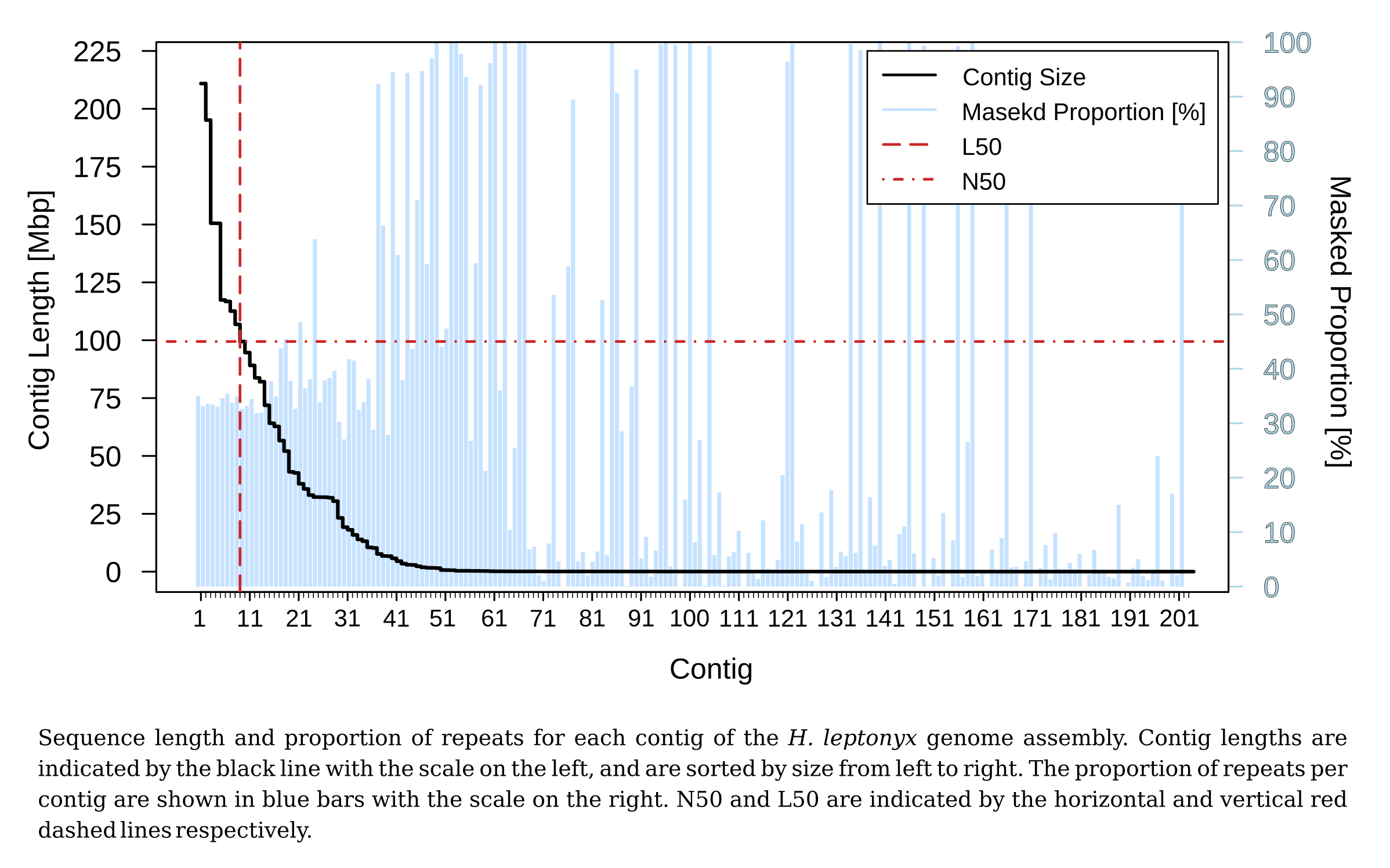

Supplement: Supplementary file 5 [file Image1.png]
