# Supplementary material for: Reference genome of the leopard seal (Hydrurga leptonyx), a Southern Ocean apex predator
Source: Front Genet. 2025 May 14;16:1561273. doi: 10.3389/fgene.2025.1561273 (PMC12118156; doi:10.3389/fgene.2025.1561273)
Supplement: Supplementary file 4 [file Supplementaryfile2.pdf]

Supplementary File S2: Results of the BUSCO analysis using the *H. leptonyx* reference genome and the lineage data set carnivora_odb10 which includes 12 genomes and 14502 BUSCOs.

```
# BUSCO version is: 5.7.0
# The lineage dataset is: carnivora_odb10 (Creation date: 2024-01-08, number of
# genomes: 12, number of BUSCOs: 14502)

# Summarized benchmarking in BUSCO notation
# BUSCO was run in mode: euk_genome_min
# Gene predictor used: miniprot

      ***** Results: *****

      C: 98.2% [S:96.1%, D:2.1%],
      F:1.1%,
      M:0.7%,
      n:14502,
      E:8.2%


      14239 Complete BUSCOs (C)     (of which 1174 contain internal stop codons)

      13935 Complete and single-copy BUSCOs (S)
      304   Complete and duplicated BUSCOs (D)
      157   Fragmented BUSCOs (F)
      106   Missing BUSCOs (M)
      14502 Total BUSCO groups searched
```

Dependencies and versions:

- hmmsearch: 3.1
- bbtools: 39.01
- miniprot_index: 0.13-r248
- miniprot_align: 0.13-r248
- python: sys.version_info(major=3, minor=9, micro=19, releaselevel='final', serial=0)
- busco: 5.7.0
