# Supplementary material for: Reference genome of the leopard seal (Hydrurga leptonyx), a Southern Ocean apex predator
Source: Front Genet. 2025 May 14;16:1561273. doi: 10.3389/fgene.2025.1561273 (PMC12118156; doi:10.3389/fgene.2025.1561273)
Supplement: Supplementary file 6 [file Supplementaryfile3.pdf]

Supplementary File S3: Summary table of the repetitive elements found in the reference genome of *H. leptonyx*. The software packages RepeatModeler 2.0.5 and RepeatMasker version 4.1.2 were used to identify different repeat families to hard-mask the genome assembly.

```
==================================================
Sequences:         241
Total length:      2455564834 bp  (2455564742 bp excl N/X-runs)
GC level:          41.60 %
bases masked:      881748612 bp ( 35.91 %)
==================================================
               number of      length   percentage
               elements*    occupied  of sequence
--------------------------------------------------
Retroelements      1010833    240303083 bp    9.79 %
   SINEs:                0            0 bp    0.00 %
   Penelope              0            0 bp    0.00 %
   LINEs:           982602    226754600 bp    9.23 %
    CRE/SLACS            0            0 bp    0.00 %
     L2/CR1/Rex      86168     23835218 bp    0.97 %
     R1/LOA/Jockey       0            0 bp    0.00 %
     R2/R4/NeSL          0            0 bp    0.00 %
     RTE/Bov-B        2074       281582 bp    0.01 %
     L1/CIN4        894360    202637800 bp    8.25 %
   LTR elements:     28231     13548483 bp    0.55 %
     BEL/Pao             0            0 bp    0.00 %
     Ty1/Copia           0            0 bp    0.00 %
     Gypsy/DIRS1         0            0 bp    0.00 %
       Retroviral    28231     13548483 bp    0.55 %

DNA transposons      40364     10443421 bp    0.43 %
   hobo-Activator    27528      6015059 bp    0.24 %
   Tc1-IS630-Pogo    12296      4160332 bp    0.17 %
   En-Spm                0            0 bp    0.00 %
   MuDR-IS905            0            0 bp    0.00 %
   PiggyBac              0            0 bp    0.00 %
   Tourist/Harbinger     0            0 bp    0.00 %
   Other (Mirage,        0            0 bp    0.00 %
    P-element, Transib)

Rolling-circles          0            0 bp    0.00 %

Unclassified:      2344916    597244037 bp   24.32 %

Total interspersed repeats:   847990541 bp   34.53 %


Small RNA:               0            0 bp    0.00 %

Satellites:              1          411 bp    0.00 %
Simple repeats:     612059     28396416 bp    1.16 %
Low complexity:     101938      5361244 bp    0.22 %
==================================================

* most repeats fragmented by insertions or deletions
  have been counted as one element


RepeatMasker version 4.1.2-p1 , default mode
run with rmblastn version 2.14.1+
```
